# The Role of Plastic Surgeons in Female Genital Mutilation Reconstructive Surgery

**DOI:** 10.29252/wjps.10.1.104

**Published:** 2021-01

**Authors:** Ava G. Chappell, Alex J. Soriano, Ivona Percec

**Affiliations:** 1 Division of Plastic and Reconstructive Surgery, Northwestern Feinberg School of Medicine, Chicago, IL, USA;; 2 Institute of Global Health, Northwestern Feinberg School of Medicine, Chicago, IL, USA;; 3 Division of Urogynecology, Department of Gynecology, University of Pennsylvania Perelman School of Medicine, Philadelphia, PA, USA;; 4 Department of Plastic and Reconstructive Surgery, University of Pennsylvania Perelman School of Medicine, Philadelphia, PA, USA.

**Keywords:** Female genital mutilation, Female circumcision, Reconstructive surgery, Plastic surgery

## Abstract

The World Health Organization defines female genital mutilation (FGM) as any procedure involving partial or total removal of female external genitalia or other injury to genital organs for non-medical indications. Despite prohibitory legislation in the United States and significant morbidity related to FGM procedures, the practice continues throughout the globe with three million women at risk annually. Surgical care for women with FGM has historically been in the hands of obstetrician and Gynaecologists (OB GYNs), and mainly focused to help safe labor and delivery. Recent awareness of the need for improved reconstructive surgical care for FGM has developed in the plastic surgical literature. This Current Opinion article highlights the historical surgical care for FGM and the opportunity for plastic surgeons to get more involved in the multidisciplinary care of these patients.

## INTRODUCTION

Female Genital Mutilation (FGM) is defined by the World Health Organization as all procedures involving partial or total removal of the female external genitalia or other injury to the female genital organs for non-medical issues.^1^^,^^2^ The complications and sequelae of FGM are immediate and long term, negatively impacting physical, psychological, and sexual health. FGM has been deemed a human rights violation by the United Nations and international human rights treaties.^3^ Despite prohibitory legislation in the United States and the significant harm related to FGM, this practice continues to persist around the globe. To date, an estimated 200 million females are living with FGM worldwide and 3 million women are at risk for FGM each year.^1^^,^^2^ In the United States, an estimated 200,000 females are currently living with FGM.^4^ This is a significant global health concern, and while prevention is the long term objective, efforts to increase community awareness and educate health care providers on diagnostic classification, long-term morbidity associated with FGM, and appropriate surgical management are warranted for multidisciplinary models of care.^4^ To improve medical care for women with FGM, improvement in reconstructive surgical technique and perioperative management must be investigated.

In the developing world, the practice of FGM varies greatly, from non-sterilized crude tools to surgical instrumentation,^5^ and the extent of FGM varies from excision of the clitoris to complete removal of the labia minora and majora with infibulation. Given the extent of surgical classifications, there is a need for reconstructive surgical care that can appropriately treat a diverse female population. Surgical reconstruction of FGM is a tool to not only restore vulvar anatomy but also regain function of the external female genitalia. Thus, the role of surgical reconstruction is critical in improving sexual function, reducing pregnancy complications, and increasing quality of life.^5^ The purpose of this paper is to encourage plastic surgeons to prioritize and understand the surgical care of women with FGM. 

***Historical Management of FGM***

Historically, surgical care for FGM patients was limited to reducing the long-term sequelae of infibulation in the setting of pregnancy and genitourinary syndromes by obstetricians and gynecologists as well as urologists. FGM can lead to near complete closure of the vaginal opening and/or vaginal scarring which poses significant risks in pregnancy as well as at the time of delivery.^6^ Thus, women with FGM were treated surgically near the time of delivery with defibulation or cesarean delivery in order to achieve childbirth without significant vulvar complications. Defibulation, which includes re-opening of the scarred vaginal introits, has also been described in the Gynaecology literature to treat dysmenorrhea, apareunia and dyspareunia.^7^

Studies on defibulation demonstrated that it is a low risk procedure with high satisfaction rates for facilitating childbirth and other long term complications from the infibulated scar.^8^

Urologists have also been involved in the reconstructive surgical care for FGM patients primarily in treating lower urinary tract symptoms associated with recurrent urinary tract infections and obstructive voiding patterns. Women with Type III FGM are at increased risk of recurrent urinary tract infections as well as urinary retention and incontinence.^9^^,^^10^

Reconstructive surgery in this patient population aims to restore vulvar anatomy in order to eliminate obstructive voiding patterns and reduce lower urinary tract symptoms. In 2012, Foldes *et al.* described the effects of FGM surgical reconstruction on female sexual function.^11^ This paper reported the results of 2,938 women with FGM type II and III treated with reconstructive surgery. Surgical correction of FGM reduced pain and improved pleasure as well as sexual health.^11^

Clitoral reconstruction has also been advocated to treat FGM by urologists and gynecologists.^12^^-^^14^ Case studies have shown that clitoral reconstruction assisted with psychosexual therapy resulted in improvement in pain with intercourse, sexual function and self-confidence.^15^ However, reconstructive techniques and accompanying psychosexual therapy protocols remain under investigation. 

***Reconstructive Surgery Options***

Currently, the available reconstructive care for FGM is not well defined and arguably insufficient.^16^ FGM survivors who seek surgical treatment present with functional symptoms, aesthetic concerns and desire to recover sexual identify.^17^ However, the satisfaction after reconstructive surgery and the experiences of these women who seek and undergo surgery is not well known.^17^^,^^18^ A recent systematic review of the literature performed by Berg *et al.*, demonstrated the current available surgical interventions for FGM are defibulation, cyst excision and clitoral reconstruction.^16^

Several current techniques are described in the following paragraphs. Defibulation is a procedure which opens a fused labia caused by infibulation scarring.^17^^,^^19^ This is typically used for treatment of FGM III, where the vaginal orifice is narrowed and/or sealed from cutting the labia minora and/or majora. The clitoris is often excised as well. Scar tissue seals the vaginal orifice and leads to urological, obstetrical, and sexual dysfunction. With the defibulation technique, the scar is released, and the vaginal orifice is re-opened. The clitoris, external urethral meatus and vulvar vestibule are also exposed.^19^

Clitoral reconstruction for women with FGM was first described by Thabet and Thabet in 2003.^20^ This technique involves release of local tissue adhesions to the clitoris, then cutting the suspensory ligament and pulling the remaining clitoris forward. The suspensory ligament is then reattached in a more posterior position. This procedure essentially exposes the remaining clitoral body.^20^

Chang *et al.*’s 2017 paper “Female Genital Mutilation Reconstruction: A Preliminary Report,” discussed an innovative clitoral restoration procedure.^21^ The procedure was performed on three patients with a history of FGM type II, who sought surgical care for sexual dysfunction and aesthetic improvement. Under conscious sedation in the outpatient setting, clitoral restoration was achieved with local scar release and dissection of the residual clitoris without release of the suspensory ligament and with the addition of labia majoraplasty. All three patients reported improved sexual function and self-confidence with their partners.^21^

The senior author (I.P) of this article has been developing a novel use of buccal mucosal grafts for reconstruction of external female gentialia after FGM. This technique involves utilizing buccal mucosa to resurface the clitoral hood after clitoral scar excision. Buccal mucosa is also used to cover the lateral aspects of medial flaps from the labia majoras designed to become the new labia minoras. Fat grafts are then harvested from the abdomen and grafted into the bilateral labia majora inferiorly and superiorly to encourage regeneration of the surgical site. At 6 months post operatively, patients have reported improved functional outcomes, while undergoing clitoral retraining therapy (unpublished data). 

***Plastic Surgery Engagement and Future Directions***

Plastic surgeons are a key part of the multidisciplinary team needed to surgically manage patients with FGM. Plastic surgery was founded on innovative techniques to restore form and function to areas of the human body after congenital and acquired defects. Arguably, no other specialty is as versed in scar release, tissue rearrangement, or painful neuroma treatment as the field of plastic surgery. In addition, plastic surgery residency training includes core competencies in: wound and burn care, tissue transfer, reconstruction of the trunk and perineum.^22^

Burn care and reconstruction surgery have been a core area of plastic surgery and represent a fundamental competency in plastic surgery residency training. The knowledge of tissue handling and scar revision procedures from burn care also can inform new methods for treating complications after FGM. While plastic surgeons’ well practiced techniques of labiaplasty and certain cosmetic genital procedures can also inform improved methods of FGM reconstruction, the essentials of plastic surgery training: tissue handling, wound care, reconstruction play a critical role as well.^22^

Physical as well as emotional and psychological trauma is a major component of FGM, and a multidisciplinary approach with psychiatrists, therapists, Gynaecologists, and plastic surgeons is needed to optimize care for FGM survivors. Post reconstruction sexual rehabilitation must also be addressed to promote recovery and improved sexual function after reconstructive surgery. Even the best technical reconstruction cannot guarantee improved function and quality of life for these women if the psychological component is not properly addressed.^22^

## CONCLUSION

Once an awareness and understanding of FGM has been realized in the plastic surgery arena, then technical innovations in clitoral and vulvovaginal reconstruction can be developed and employed. However, this does not mean to depart from the techniques already reported in the gynecologic and urological literature, but rather, expand upon these techniques and work with Gynaecologists and urologists who have historically cared for these patients. Plastic surgeons are accustomed to collaborative environments and need not stand alone but join with the others who have been working to help FGM survivors regain the rights to their body, sexuality, and lives. 
